# A simple blinding index for randomized controlled trials

**DOI:** 10.1016/j.conctc.2024.101393

**Published:** 2024-11-26

**Authors:** David Petroff, Miroslav Bacak, Nikolaos Dagres, Patrick Dilk, Rolf Wachter

**Affiliations:** a Clinical Trial Centre, Faculty of Medicine, Leipzig University, Germany; b Department of Cardiology, Angiology and Intensive Care Medicine, Deutsches Herzzentrum der Charité, Berlin, Germany; c Department of Electrophysiology, Heart Center Leipzig, Germany; d Department of Cardiology, University Hospital Leipzig, Germany

**Keywords:** Blinding, Blinding index, Randomized controlled trial, Statistical test, Wilson score method

## Abstract

Blinding is an essential part of many randomized controlled trials. However, its quality is usually not checked, and when it is, common measures are the James index and/or the Bang index. In the present paper we discuss these two indices, providing examples demonstrating their considerable weaknesses and limitations, and propose an alternative method for measuring blinding. We argue that this new approach has a number of advantages. We also provide an R-package for computing our blinding index.

## 1 Introduction

Consider a randomized controlled trial (RCT) with two allocation groups, A and B. Each trial participant is randomly allocated to one of these groups. We consider the situation in which concerted effort is made to conceal the result of allocation, i.e. certain individuals should ideally not know if a given trial participant is in group A or B. These individuals are commonly the trial subjects themselves or the trial investigators, but may also be endpoint assessors. This procedure is referred to as *blinding.* We do not consider the rare situation in which the subjects are deliberately misled into believing that they are in the non-allocated group. The importance of blinding stems from the fact that knowledge of the assigned group may influence the results of the trial, for instance through the placebo effect or by affecting endpoint assessment. It is therefore important to check whether the blinding was successful. Unfortunately, in most RCTs the quality of blinding is not measured at all [1], [2], [3].

To acquire data on the quality of blinding, individuals may be asked in which allocation group they believe the trial subjects are, where possible responses may include the option ‘I don’t know’. The frequencies of their responses are recorded using the following notation:

(1) $$\begin{array}{ccc} \mathrm{Answer} & \mathrm{GroupA} & \mathrm{GroupB}\\ \text{I’m in A} & n_{AA} & n_{AB}\\ \text{I’m in B} & n_{BA} & n_{BB}\\ \text{I don’t know} & n_{DA} & n_{DB} \end{array}$$

James et al. [4] introduced a sophisticated blinding index, which has since been called the *James blinding index,* for evaluating the data in (1). Later Bang et al. [5] came up with an allocation group specific blinding index. This is called the *Bang blinding index.* Both the James and Bang indices have become standard measures for evaluating the success of blinding in RCTs, when it is measured at all [1]. One of our main goals in the present paper is to discuss these two indices in various scenarios and demonstrate that one should exercise caution when using or interpreting them. To this end, we present several examples of answer distributions (following the pattern (1)) in Section 2 below and compute the corresponding indices. It turns out that the interpretation is sometimes rather difficult since the index values do not correspond to our intuitive understanding of successful blinding nor, perhaps, with what one intends to measure.

To overcome these difficulties, we propose a very simple index for measuring the quality of blinding as an alternative; see Section 3. Our method has the following advantages over James and Bang:

(i) It is conceptually simple.
(ii) It is mathematically simple.
(iii) It relies only on the intrinsic symmetry of successful blinding.
(iv) It requires no additional instructions for the subjects (like “You should answer ‘I don’t know’ only when…”).
(v) It applies even if randomization is not in a 1:1 ratio.

Using data from double-blinded trials, we show in Section 4 that the weaknesses discussed from a theoretical perspective are also relevant problems arising in clinical research. In Section 5 we introduce the SBI (“simple blinding index”) R-package [6], which provides an estimate for the new blinding index along with a confidence interval and a p-value dual to it.

Our computations of the James and Bang blinding indices for the purposes of the present paper rely on the R-package BI [7], whose existence we gratefully acknowledge.

## 2 Discussion of the existing methods

### 2.1 “I don’t know” answers

We quote from James et al. [4, p. 1430]:

“The blindness index proposed in this paper assumes that the most desirable response is ‘don’t know’. If the respondents detect that this is the most socially acceptable response, they may provide this answer even though they really think that the patient received a specific study treatment. Therefore, the key to blindness assessment is to elicit the information so that the respondent provides an honest accounting of which treatment s/he thinks the patient received. One should encourage respondents to answer ‘don’t know’ only if they truly do not know or cannot make an educated guess”.

Indeed, one difficulty in interpreting the answer “I don’t know” is that the probability of choosing it does not depend primarily on the success of blinding, but on the precise formulation of the question posed and the interaction between questioner and respondent. A real-world example of this phenomenon is presented in Section 4.1. Comparing our example to Table A13 at post-randomization from the ORBITA trial [8] shows that the proportion of patients providing the answer “I don’t know” can vary widely from 60% in our case to 99% in theirs although we would argue that blinding is equally good. Note also that the “I don’t know” answers are understood differently by James and Bang, as discussed in [1].

In general, we recommend not to allow the answer “I don’t know” at all. If the individuals have no idea about the trial subjects’ allocation, they should simply pick one of the trial groups at random.

### 2.2 The James index

The James index ranges from 0 and 1. If all subjects guess their group allocation correctly, the James index is equal to 0. The value of 1 is reached either if all subjects respond “I don’t know” or if all guess the opposite of the truly allocated group, see Table 1.

This implies that these two situations are equally desirable, which we do not agree with on two counts. In the first case, we believe that the proportion of subjects who choose “I don’t know” can be increased by suggesting this be chosen unless they are quite sure of the allocation. In the second, we believe that misleading subjects to chose the wrong group is rarely the desired outcome of blinding. As a result of these definitions, the James index reflects improved blinding as one proceeds from the value 0 to 1/2, but between 1/2 and 1, it is difficult to interpret if there is any improvement in blinding. In that sense it cannot be considered a single index.

**Table 1:** The James index attains its maximum of 1 in both scenarios depicted below. While the left-hand side case could certainly be interpreted as perfect blinding, the right-hand one is not what is generically intended with blinding.

Answer	Group A	Group B	Answer	Group A	Group B
I’m in A	0	0	I’m in A	0	100
I’m in B	0	0	I’m in B	100	0
I don’t know	100	100	I don’t know	0	0

Other illustrative examples for the James index are presented in Table 2, Table 3.

The first example depicts two scenarios which both lead to a James index of 0.5 with the same confidence interval (CI) despite the fact that the first implies excellent blinding and the second very poor blinding. In the second example, the blinding obviously did not work well, but the James index is relatively high and its CI narrow.

**Table 2:** The James index is in both scenarios equal to 0.5 with 95%-CI (0.43,0.57), even though we, based on our intuitive understanding of successful blinding, consider the left-hand case significantly more blinded.

Answer	Group A	Group B	Answer	Group A	Group B
I’m in A	50	50	I’m in A	50	0
I’m in B	50	50	I’m in B	0	50
I don’t know	0	0	I don’t know	50	50

**Table 3:** Responses leading to the James index of 0.85 with 95%-CI (0.80,0.90).

Answer	Group A	Group B
I’m in A	15	0
I’m in B	0	15
I don’t know	85	85

**Table 4:** The Bang index does not work when the probability of choosing arm A does not equal that of arm B. For the table on the left, one finds 0.36 with 95%-CI (0.17,0.54) for arm A and, −0.36 with 95%-CI (−0.63,−0.10) for arm B. For the table on the right, one finds 0.49 with 95%-CI (0.28,0.70) for arm A and −0.52 with 95%-CI (−0.81,−0.22) for arm B.

Answer	Group A	Group B	Answer	Group A	Group B
I’m in A	36	18	I’m in A	50	25
I’m in B	12	6	I’m in B	17	8
I don’t know	19	9	I don’t know	0	0

The above examples show, that the James blinding index does not correspond to our intuitive understanding of successful blinding and we are therefore not convinced that it is an appropriate measure.

Furthermore, when using the James index, one has to choose weights for correct, incorrect and “I don’t know” answers. While such a weight assignment makes the James index more flexible, its obvious disadvantage is that there is no standard way of choosing the weights and the resulting index is therefore more difficult to interpret and/or compare.

### 2.3 The Bang index

The Bang index is allocation group specific, that is, there are actually two Bang indices ${\mathrm{Bang}}_{A}$ and ${\mathrm{Bang}}_{B}$ for the group A and group B, respectively. They can be estimated by

(2) Bang^X≔(2nXXnAX+nBX−1)nAX+nBXnAX+nBX+nDX,

where X∈{A,B} stands for the allocation group; see [5, Eq. (1)]. It is easy to see that the Bang index (as well as its estimator above) ranges from −1 to 1. The value 0 is considered ideal.

The first issue we encounter when applying the formula in (2) occurs when all subjects answer “I don’t know”, that is, when $n_{AX}=n_{BX}=0$. The right-hand side is then not well-defined (because of division by 0) and consequently the R-package BI (version 1.2.0) returns NaN (“Not a Number”). Fortunately, there is an easy fix to this problem, one can rewrite the formula in (2) equivalently as

(3) Bang^X=2nXX−nAX−nBXnAX+nBX+nDX,

where division by 0 cannot occur.

However, there is a far more serious problem regarding the Bang index. Namely, it assumes that given perfect blinding, the probability of choosing arm A is equal to the probability of choosing arm B, implying that $n_{AX}\approx n_{BX}$. There is no reason to expect this if randomization is not 1:1, but even if it is, the probability of choosing the intervention arm could depend on the subject’s prior belief regarding its superiority coupled with general attitudes regarding luck, superstition, optimism, pessimism, being a frequentist/Bayesian, etc. Two examples of the resulting Bang indices in such situations are presented in Table 4.

**Table 5:** A comparison of the three indices for the examples provided in this paper. Whereas the James and Bang indices allow for the answer “I don’t know”, our index does not. For comparison purposes, our index was calculated by dividing the “I don’t know” answers equally between the groups A and B. The James index has the value 1 when all individuals answer “I don’t know” and values between 0.5 and 1 indicate acceptable blinding. Perfect blinding for the other two indices has the value 0. NaN stands for “Not a Number” and comes from the division by 0 discussed in Section 2.

Table	James index		Bang index, A		Bang index, B		Our index	
	Estimate	95%-CI	Estimate	95%-CI	Estimate	95%-CI	Estimate	95%-CI
Table 1 left	1.00	(1.00,1.00)	NaN	(NaN,NaN)	NaN	(NaN,NaN)	0.00	(−0.14,0.14)
Table 1 right	1.00	(1.00,1.00)	−1.00	(−1.00,−1.00)	−1.00	(−1.00,−1.00)	−1.00	(−1.00,−0.95)
Table 2 left	0.50	(0.43,0.57)	0.00	(−0.20,0.20)	0.00	(−0.20,0.20)	0.00	(−0.14,0.14)
Table 2 right	0.50	(0.43,0.57)	0.50	(0.40,0.60)	0.50	(0.40,0.60)	0.50	(0.37,0.61)
Table 3	0.85	(0.80,0.90)	0.15	(0.08,0.22)	0.15	(0.08,0.22)	0.15	(0.01,0.28)
Table 4 left	0.64	(0.55,0.73)	0.36	(0.17,0.54)	−0.36	(−0.63,−0.10)	−0.00	(−0.18,0.19)
Table 4 right	0.51	(0.41,0.60)	0.49	(0.28,0.70)	−0.52	(−0.81,−0.22)	−0.01	(−0.17,0.18)

**Table 6:** Comparison of two trial sites of the PVI-Sham-AF study as of March 7, 2024.

Patient’s answer	Trial site 1	Trial site 2
I’m in the intervention group	17(61%)	15(21%)
I’m in the sham group	3(11%)	5(7%)
I don’t know	8(29%)	52(72%)

## 3 Our method

### 3.1 Philosophical background

We believe that the purpose of blinding is to exclude the causal impact of knowledge of allocation on outcomes. On the other hand, we do not require that successful blinding affect the belief regarding the group that subjects were allocated to. Therefore, we claim that blinding is successful when there is symmetry between arms, i.e. the subjects in arm A and those in arm B have identical beliefs. We see no evidence for lack of successful blinding if, say 90% of subjects believe they are allocated to group A, so long as this does not depend on the true allocation group.

We understand the reasoning of Bang et al. [5] about the importance of the allocation group specific blinding index, however our purpose is to introduce a blinding index which measures the quality of blinding in the entire trial under consideration and possibly indicates its failure. For this reason, our index is not allocation arm specific.

When assessing the success of blinding, we do not believe it is informative to allow “I don’t know” answers. As noted above, the meaning of this answer can change from person to person and site to site and they can distort the results, see also Section 4.1. Subjects who have no idea which group they are in will select one at random.

Another crucial aspect is timing. Shortly after randomization, successful blinding implies that patients in groups A and B have the same beliefs, but this need not necessarily hold once the effects of the intervention become palpable. Regardless of the method, it is important to pose the question at the right moment. We agree with Hemila [9] and Henneicke-von Zepelin [10] that this should be shortly enough after the randomization so that the responses are not influenced by the therapy effect. Furthermore, it should be sufficiently long after treatment that an information leak, should it have occurred, can be measured.

### 3.2 Description of our method

Building upon the premises from Section 3.1, we now introduce a very simple statistical index and test for detecting a failure in the blinding of an RCT. Let us consider a scenario described in (4).

(4) $$\begin{array}{ccc} \mathrm{Guess} & \mathrm{GroupA} & \mathrm{GroupB}\\ \text{I’m in A} & n_{AA} & n_{AB}\\ \text{I’m in B} & n_{BA} & n_{BB} \end{array}$$

Without loss of generality, we consider the probabilities of guessing that trial subjects are in group A. These are denoted by $p_{A}$ and $p_{B}$ for subjects who are actually in group A and B, respectively. We estimate these two probabilities by

(5) pA^≔nAAnAA+nBA,pB^≔nABnAB+nBB.

As a blinding index, we simply take the difference $p_{A}-p_{B}$, which ranges from −1 to 1, and estimate it by pA^−pB^. Based on the symmetry argument above, successful blinding implies the null hypothesis $p_{A}=p_{B}$, meaning that the value 0 for the our blinding index implies perfect blinding.

The index is trivial to interpret, since it is simply the difference in probabilities for guessing group A between the real arms. To construct a CI, we use the Wilson score method proposed by Newcombe [11, Eq. (10)]. Given a CI, we also find the corresponding p-value. Note that while the $\chi^{2}$-test is certainly appropriate for testing the frequency differences in (4), our purpose is to introduce an *index* as a *quantitative* measure of the quality of blinding.

In Table 5 we compare indices between the methods for the tables provided above. A direct comparison is complicated by two main issues. For one, James and Bang allow the answer “I don’t know” and for another, the scale used by James is not easily interpretable between the values of 0.5 and 1, as discussed above. However, qualitatively contradictory statements based on the indices constitute a problem and we focus here on such cases.

For comparison purposes, our index was calculated by dividing the “I don’t know” answers equally between the groups A and B. The comparison shows that the James index disagrees with ours in rows 2, 4 and 5. Row 2 demonstrates that the James index fails to distinguish between perfect blinding and the situation in which every individual provides the wrong arm as an answer. Rows 4 and 5 show that the James index is not particularly sensitive to correct “guessing” if enough “I don’t know” answers are mixed in. The Bang index disagrees fundamentally with ours in rows 6 and 7, since the number choosing arm A differs from the number choosing arm B. Such situations are expected to arise when allocation is not in a 1:1 ratio or in situations in which all patients favor a given answer such as in the examples of acupuncture trials below. Note however, that as long as individuals from arm A do not differ in their answers from those in arm B, there is no reason to suspect that the blinding process as defined in Section 3.1 was faulty. Both the James and Bang indices have the well-known problems with estimates of variance and hence the width of CIs as probabilities approach 0 or 1, i.e. in rows 1 and 2.

If one wishes to compute our index from data with “I don’t know” answers allowed, we recommend to proceed like in our comparison in Table 5. However, this is arguably not the only option.

## 4 Real world examples

### 4.1 Problems with the answer “I don’t know”

Our interest in measuring the success of blinding in RCTs originated from our work on the currently recruiting “PVI-Sham-AF” trial (registration number NCT05119231) headed by the two co-authors ND and RW and coordinated by the co-author PD. In that trial, the patients are randomly allocated to the intervention and control groups in a 2:1 ratio and remain blinded with respect to the allocation for the entire follow-up period. To measure the quality of blinding, the patients are asked immediately after the intervention/sham procedure in which group they believe they are. Possible answers are “I’m in the intervention group”, “I’m in the sham group” and “I don’t know”. In Table 6 we see the differences in answer frequencies in the two trial sites with the strongest recruitment, 8/28 (29%) answered “I don’t know” in one site vs. 52/72 (72%) in the other.

We see this as clear evidence that the “I don’t know” answer frequency strongly depends on the interaction between the medical staff and patients alluded to in Section 2. The patients in the PVI-Sham-AF trial also answer additional questions concerning their blinding (like “Is your answer based on a certain piece of information, or is it just an assumption?”), so that we get more insight into their way of thinking. It turns out that patients may consider things to be “certain pieces of information” that are actually mere guesses or improbable interpretations of signals. Note that blinding indices cannot be provided for this example until completion of the trial and the subsequent unblinding.

### 4.2 Comparison of the three indices using data from clinical trials

Now we would like to discuss the blinding indices using real clinical trial data. To this end we consider selected trials in acupuncture research from [12, Table 3] demonstrating exemplary similarities and differences. The results are summarized in Table 7.

The first two rows in Table 7 provide an example of agreement between all three methods that blinding was successful. Likewise the examples from rows 3 and 4 in Table 7 are obviously very poorly blinded, as confirmed by all the indices. The following two examples (rows 5 and 6 in Table 7) are perhaps most interesting since they show trials where blinding worked well according to our definition, whereas the Bang indices claim that blinding was unsuccessful. For example, in the trial by Lee et al. [17] the number of patients who guessed acupuncture vs. sham was 33 vs. 11 in the acupuncture arm and 36 vs. 9 in the sham arm. Although the majority of patients believed they were in the acupuncture arm, the guesses did not depend on the true arm, hence we claim that blinding was successful. The last row in Table 7 illustrates additional problems with the Bang index, namely that the absolute values of the confidence intervals can exceed 1 and that a zero in the data results in a confidence interval of zero width.

**Table 7:** A comparison of the blinding indices for data from real clinical trials. The James index has the value 1 when all individuals answer “I don’t know” and values between 0.5 and 1 indicate acceptable blinding. Perfect blinding for the other two indices has the value 0.

Reference	James index		Bang index, A		Bang index, B		Our index	
	Estimate	95%-CI	Estimate	95%-CI	Estimate	95%-CI	Estimate	95%-CI
Zaslawski [13]	0.78	(0.68,0.88)	−0.12	(−0.37,0.13)	−0.03	(−0.28,0.21)	−0.08	(−0.30,0.16)
Shin [14]	0.66	(0.52,0.81)	−0.05	(−0.41,0.31)	0.05	(−0.29,0.38)	0.00	(−0.28,0.28)
Chae [15]	0.18	(0.04,0.32)	0.57	(0.14,1.00)	0.71	(0.35,1.08)	0.64	(0.28,0.82)
Takakura [16]	0.31	(0.23,0.39)	0.60	(0.40,0.80)	0.17	(−0.08,0.42)	0.38	(0.21,0.53)
Lee [17]	0.52	(0.44,0.61)	0.50	(0.24,0.76)	−0.60	(−0.83,−0.37)	−0.05	(−0.22,0.12)
Streitberger [18]	0.47	(0.39,0.54)	0.87	(0.69,1.05)	−0.73	(−0.98,−0.49)	0.07	(−0.10,0.24)
Tong [19]	0.44	(0.36,0.52)	1.00	(1.00,1.00)	−0.81	(−1.06,−0.56)	0.10	(−0.01,0.29)

## 5 How to compute our index in R

To compute our blinding index, one can use our R-package ‘SBI’, which stands for a “Simple Blinding Index” [6]. Assuming the situation depicted in (1), the following piece of code

BlindingIndex(n_AA, n_BA, n_AB, n_BB, conf.level =0.95)

yields the desired estimate as well as its 95% confidence interval as defined in Section 3 above. It also provides a p-value dual to the Wilson confidence interval used in this routine.

## 6 Concluding remarks

We have argued that the commonly used James and Bang indices are inadequate in many cases and have introduced a simple alternative that remedies them. It no longer permits “I don’t know” as an answer to the question “Which arm do you believe you are in?” In contrast to the Bang index, ours does not provide arm specific information, which can be regarded both as a limitation and a necessity. It is a limitation when there is good reason to require that respondents from each arm guess arms A and B with equal probability. In general, however, this assumption will not hold, as shown in the acupuncture examples above, and blinding can only be assessed by considering both arms together. Another potential limitation with our new index is that patients unwilling to a make a random guess, but not provided with the option “I don’t know”, may simply not respond and hence generate missing data. Finally, we do not see an easy way to generalize our index for multi-arm trials. This would be, in our opinion, an interesting topic for future research.

## CRediT authorship contribution statement

**David Petroff:** Writing – review & editing, Writing – original draft, Supervision, Software, Methodology, Investigation, Formal analysis, Conceptualization. **Miroslav Bacak:** Writing – review & editing, Writing – original draft, Software, Formal analysis, Conceptualization. **Nikolaos Dagres:** Writing – review & editing, Methodology, Conceptualization. **Patrick Dilk:** Writing – review & editing, Methodology, Conceptualization. **Rolf Wachter:** Writing – review & editing, Methodology, Conceptualization.

## Declaration of competing interest

The authors declare that they have no known competing financial interests or personal relationships that could have appeared to influence the work reported in this paper.
